# Idiopathic Intracranial Hypertension in Children and Adolescents with Obesity: A Narrative Review

**DOI:** 10.3390/children13010001

**Published:** 2025-12-19

**Authors:** Nicola Improda, Giada Ballarin, Selvaggia Lenta, Laura D’Acunto, Celeste Tucci, Marta Giovengo, Claudia Mandato, Antonio Varone, Maria Rosaria Licenziati

**Affiliations:** 1 Neuro-Endocrine Diseases and Obesity Unit, Department of Neurosciences, Santobono-Pausilipon Children’s Hospital, Via Egiziaca a Forcella 18, 80139 Naples, Italy; g.ballarin@santobonopausilipon.it (G.B.); m.licenziati@santobonopausilipon.it (M.R.L.); 2 Childhood Cancer Registry of Campania, Santobono-Pausilipon Children’s Hospital, 80129 Naples, Italy; s.lenta@santobonopausilipon.it; 3 Pediatric Neurology Unit, Department of Emergency, Santobono-Pausilipon Children’s Hospital, 80129 Naples, Italy; l.dacunto@santobonopausilipon.it (L.D.); c.tucci@santobonopausilipon.it (C.T.); a.varone@santobonopausilipon.it (A.V.); 4 Department of Medicine, Surgery and Dentistry “Scuola Medica Salernitana”, University of Salerno, 84081 Baronissi, Italy; m.giovengo@studenti.unisa.it (M.G.); cmandato@unisa.it (C.M.); 5 Chronic Diseases, Hepatology and Nutrition Unit, Santobono-Pausilipon Children’s Hospital, 80129 Naples, Italy

**Keywords:** idiopathic intracranial hypertension, obesity, weight loss, refractory headache, cerebrospinal fluid, papilledema

## Abstract

**Highlights:**

**What are the main findings?**

- The increasing incidence of IIH in children and adolescents is strongly linked to the global obesity epidemic.
- Weight loss (6–10%) remains the cornerstone of treatment, with bariatric surgery and new anti-obesity medications (GLP-1 agonists) emerging as highly effective options for long-term IIH remission.

**What is the implication of the main finding?**

- The complex pathophysiology—linking obesity, hormones, and CNS pressure—mandates a multidisciplinary team (Neuro-Ophthalmology, Pediatric Endocrinology, Nutrition) for optimal patient prognosis.
- Since weight loss is the only etiological treatment, therapeutic strategies must prioritize prompt, effective weight reduction, incorporating newer anti-obesity agents and surgical options in refractory adolescent cases to prevent permanent vision loss.

**Abstract:**

Background: Idiopathic intracranial hypertension (IIH), also known as primary pseudotumor cerebri, is characterized by increased intracranial pressure (ICP) without an identifiable cause. It can lead to significant morbidity, including permanent vision loss, especially in younger children. The exact cause of IIH is still unclear, but excess adiposity seems to be a key risk factor. Current treatment options are unsatisfactory, but research is exploring novel therapies targeting obesity-related mechanisms. Methods: Narrative review of the literature aimed at summarizing current knowledge regarding the epidemiology, pathophysiology, clinical features, treatment options and long-term outcomes for pediatric IIH, with a particular focus on the link with obesity. Results: The incidence of IIH is rising, mirroring the obesity epidemic. Excess adiposity, predominantly visceral, might cause IIH through several factors such as decreased venous return, hormone dysregulation, inflammation, obstructive sleep apnea, and dysfunction of the glymphatic system. The extent of weight loss required and the most appropriate strategy to achieve it are still uncertain. Given the difficulty in achieving and maintaining weight loss with dietary strategies, bariatric surgery and weight loss medications are emerging as effective options for long-term remission of both obesity and IIH. Conclusions: IIH is a rare and poorly understood disease. At present, weight loss represents the only treatment that addresses the pathophysiology of IIH. The role and potential as standalone or synergistic therapies of weight loss drugs and bariatric surgery for IIH in adolescents require future research.

## 1. Introduction

Idiopathic intracranial hypertension (IIH), also known as primary pseudotumor cerebri, is characterized by increased intracranial pressure (ICP) without any identifiable cause. The diagnosis of IIH is based on the demonstration of an opening pressure of the cerebrospinal fluid (CSF) > 25 cm H_2_O by lumbar puncture, in the absence of organic brain lesions (i.e., hydrocephalus or space-occupying lesion), abnormal CSF composition or systemic diseases [1,2]. Its aetiology and pathophysiology are still to be elucidated, but a striking association with obesity has been observed [3,4]. Central obesity is thought to contribute to IIH through several interconnected pathways, including mechanical, hormonal and inflammatory factors [5,6]. Although previously defined as benign, if not appropriately treated, IIH is associated with significant morbidity, due to disease recurrence and/or the risk of permanent visual loss and/or sixth or seventh nerve palsy [1,7]. Children can be exposed to a higher risk of severe complications than adults, likely due to the inability to report specific symptoms or the possible asymptomatic course of the disease [8]. The treatment of IIH remains a subject of considerable clinical debate and requires a comprehensive, multidisciplinary approach [8]. While weight loss is recognized as a key factor in disease recovery, the specific amount of weight loss needed and the most effective strategy to achieve it are not yet fully understood [1,2]. Bariatric surgery and weight loss medications are emerging as promising, effective treatments for IIH [1,2]. They can be used as standalone or synergistic therapies; however, evidence for their efficacy, particularly in adolescents, remains limited.

This narrative review aims to synthesize current research on the link between obesity and IIH in children and adolescents. We also outline the distinct clinical characteristics of the disease in obese subjects, providing a theoretical framework to guide the treatment of this uncommon condition.

## 2. Materials and Methods

A comprehensive literature search, structured around the PEO framework (Population: children and adolescents of any ethnicity; Exposure: overweight or obesity; Outcome: diagnosis of idiopathic intracranial hypertension), was performed. Initial searching of the PubMed and Cochrane databases was limited to papers written in English over the previous 25 years. Selected keywords included: ‘Idiopathic Intracranial Hypertension’ OR ‘Pseudotumor cerebri’ OR ‘Benign Intracranial Hypertension’ AND (‘Paediatric’ OR ‘Adolescent’ OR ‘Children’) AND (‘Overweight’ OR ‘Obesity’).

The selection process is fully detailed as follows: after the removal of 8 duplicates, 286 articles were screened based on title and abstract, with primary exclusions due to not meeting the PEO criteria. Case reports were considered only when no other article type was available for a specific topic. Following the exclusion of 110 articles, the remaining 176 full texts were assessed for eligibility, and further studies were excluded based on a failure to provide primary data or an irrelevant study design. Additional relevant publications were identified through a manual search of the bibliographies. Eventually, 133 studies were prioritized based on clinical relevance, study design, recentness, and a high number of participants.

## 3. Epidemiology of IIH

The incidence of IIH in the general population is increasing over the years, rising from 0.5 to 2/100,000 people per year in 2002 to 4.69/100,000 people per year in 2016 [9]. This rise appears to mirror the increasing prevalence of obesity [9], as also confirmed by a meta-analysis of 15 population-based studies involving subjects older than 14 years [10]. Indeed, more than half of the cases of IIH occur in obese women aged 14–45 years, with a peak incidence at 25 years [9,10].

Large epidemiological studies evaluating the incidence of IIH specifically in the pediatric population are currently lacking. The results of the studies assessing the epidemiology of pediatric IIH [8,9,10,11,12,13,14,15,16,17,18,19,20,21,22,23] are summarized in Table 1, in chronological order of publication.

Despite the faster increase in obesity rates compared to adults [16], the overall incidence of pediatric IIH remains slightly lower, at 0.5–1.2 per 100,000 individuals [17,18,19]. However, subjects aged 12–17 years are affected at an incidence rate much higher than that of those aged 2–12 years (0.8 to 8.69 vs. 0.16 to 1.16 per 100,000 children/year, respectively) [7,19,20,21,24]. A recent paper, covering United States data between 1990 and 2024, reported progressively increasing incidence rates of IIH, both in children and adolescents [24] (Table 1). Despite being reported in all ethnic groups, up to 90% of the cases have been found in non-Hispanic White children [23]. Familial cases are infrequent, with only about 0.03 percent of the patients having a family member with IIH in the same study [23].

## 4. Clinical Presentation of IIH

IIH is clinically characterized by signs and symptoms of increased ICP (headaches, nausea, vomiting, transient obscurations of vision, papilledema) and no localizing neurological signs, with the only exception being unilateral or bilateral VI nerve paresis [25,26,27]. Less commonly pediatric patients with IIH may present with pulsatile tinnitus, stiff neck, dizziness, cognitive disturbances, irritability (more likely to present in infants and young children), and cranial neuropathies [8,22,28,29]. Clinical features differ among pre- and post-pubertal subjects, with the former remaining asymptomatic on early presentation in up to 29% of the cases [30], while the latter showing a higher tendency to be symptomatic [11,22,31,32], overlapping with adult patients [22,33]. Consistent findings across multiple studies [34,35,36,37,38] indicate that overweight or obese children at the onset of IIH have more symptoms than their normal weight counterparts. In addition, despite contrary results [9,39,40,41,42], there is evidence that BMI correlates with the risk of serious complications such as visual loss or headache chronification [41,43], as well as with the risk of recurrence of IIH [31]. Headache represents the most common presenting symptom reported among studies (up to 91% of patients), particularly in late childhood and adolescence [8,22,38]. Obese children/adolescents frequently report headaches, particularly migraine, thus making early recognition of IIH challenging [44,45]. Features suggestive of IIH, albeit non-specific, may be headache duration > 2 h, insufficient pain relief with non-steroidal anti-inflammatory drugs, avoidance of playing, being aggravated by leaning forward, retrobulbar pain, and pain with eye movement [8,28,46,47,48]. Obese children and adolescents presenting with IIH also exhibit higher rates of concomitant arterial hypertension and liver steatosis, in comparison to patients with obesity without IIH [46].

## 5. Diagnosis of IIH

According to 2013 Friedman’s criteria, the diagnosis of IIH is based on three fundamental points: the detection of papilledema, CSF hypertension demonstrated by a lumbar puncture, and neuroradiological signs suggestive of intracranial hypertension [49]. The diagnosis of IIH is considered definite in the presence of papilledema and/or abducens nerve palsy plus raised CSF pressure, probable when papilledema is associated with normal CSF pressure, or finally “suggestive of” IIH when even in the absence of papilledema and abducens nerve palsy there are at least three valid neuroimaging markers of raised ICP, among empty sella, flattening of the posterior aspect of the ocular globe, distension of the perioptic subarachnoid space with or without tortuous optic nerve, and transverse venous stenosis [49].

Papilledema can be detected incidentally and is considered the single most important predictor of visual loss in these patients [50,51]. Its severity is defined in the fundoscopy by the Frisen scale, in which stages 0–1 are considered low-risk stages, and from grade 2 onwards, the risk of ICP is high [8,52]. In doubtful cases, fundus oculi can be coupled with optical coherence tomography (OCT) and ultrasound of the optic nerves, which represent non-invasive reliable tools differentiating between true papilledema and pseudo-papilledema [53,54,55,56,57]. Indeed, OCT-derived peripapillary retinal nerve fiber layer (RNFL) thickness in cases with raised ICP is significantly higher than pseudo-papilledema and correlates with CSF opening pressure (OP) [58].

The cut-offs for CSF-OP in obese subjects remain debated [59,60,61,62,63,64]. While some earlier investigations reported no correlation [63,64], recent evidence, including the findings reported by Çağ et al. (2024) [62], continues to demonstrate a positive relationship between BMI and CSF-OP in specific subgroups of children [60,61,62]. According to the Friedman criteria, CSF-OP is considered pathological above 280 mm CSF in obese children, or above 250 mm CSF if the child is not sedated and not obese [49].

## 6. Pathophysiology Underlying the Association Between IIH and Obesity: A Roadmap to Novel Treatments

**Figure 1:**
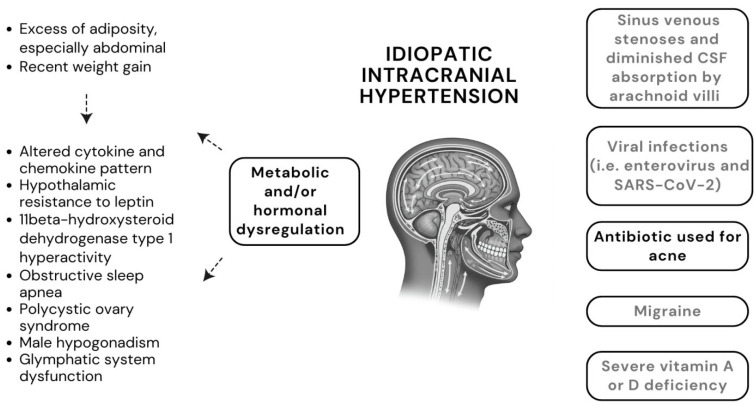
Pathogenic mechanisms proposed for idiopathic intracranial hypertension (grey boxes represent unlikely mechanisms, based on current knowledge). The figure and any element included are free from any copyright.

The pathophysiology of IIH still remains elusive. Several mechanisms have been proposed so far (Figure 1). While the rationale for several factors, including viral infections, migraine, deficiency in vitamin A and D, is very weak, metabolic and/or hormonal imbalance secondary to excess adiposity represents the most compelling candidate mechanism (summarized in Table 2) [11,34,35,65,66,67]. In this context, although sinus venous stenoses are frequently detected in patients with IIH, these are more likely to represent a consequence rather than a cause of IIH, since they may resolve after reduction of ICP [11].

Overweight or obesity has been reported as the sole risk factor for IIH in about half of the patients, while the others had at least one co-occurring medical condition, especially migraine or antibiotic use for acne [11]. A strong association was also observed between increasing weight class and IIH in adolescents, with progressively increasing adjusted odds ratios: 1.00 for underweight/normal weight, 3.23 for overweight, 4.29 for moderately obese, and 15.37 for extremely obese subjects [67]. Accumulating evidence [11,34,35,65,66], including a study on 1499 subjects [35], indicates notable differences in IIH presentation across age groups. Specifically, young children had lower rates of female predominance (20%) and obesity (10%) compared to adolescents (82% female, 64% obese) and adults (85% female, 80% obese) [35]. However, the question of whether these age-related groups are points along a disease continuum or represent distinct types of pediatric IIH with different clinical and pathophysiological profiles remains open.

While early studies using anthropometric indices of body fat distribution indicated increased lower-body fat [15], other studies relying on dual-energy X-ray absorptiometry (DXA) scanning showed that patients with IIH have predominantly abdominal fat deposition. This latter type of fat correlates with lumbar puncture opening pressures, whereas BMI does not [68]. A key pathogenic role of abdominal fat is also suggested by the fact that patients who experience a decrease in ICP following therapeutic weight loss exhibit a preferential reduction in truncal fat rather than subcutaneous fat [68]. It has been hypothesized that central obesity might increase pressure within the abdomen, chest cavity, and central veins, potentially leading to higher cerebral venous pressure and, eventually, to raised ICP [69]. However, this hypothesis does not explain why IIH is more common in women, occurs in normal weight individuals, and is not more prevalent in pregnant women. In this regard, there is evidence in children [70] and young adults [69,71] that the speed of weight gain, not the ultimate weight, is the key factor triggering IIH. Indeed, a moderate weight gain (5% to 15%) in the year prior to diagnosis increases the risk of IIH in both obese and non-obese young adults [40,41,68,72]. Adolescents with IIH also have height Z-scores higher than age- and gender-matched reference standards [34], prompting the hypothesis that accelerated growth and IIH may share common mechanisms in overweight and obese youth [23].

In particular, alterations in sex hormones, especially androgens, may play a central role in the pathophysiology of IIH [73,74]. Experimental data indicate that testosterone can enhance CSF secretion [74], possibly by acting through androgen receptors and the androgen-activating enzyme aldo-keto reductase family 1 member C3 (AKR1C3) expressed in the choroid plexus [74]. Excess androgens in obese adolescents may stem from an exacerbation of the transient anovulatory cycles and hyperandrogenemia commonly seen at their age [75,76]. Furthermore, women with IIH exhibit a higher prevalence of polycystic ovary syndrome (PCOS) compared to those with simple obesity (57% vs. 28.3%) [77], with androgen levels also correlating with a younger age of diagnosis [68]. Finally, recent metabolomics data revealed that women with IIH exhibit an androgen profile distinct from PCOS and obesity, characterized by elevated serum testosterone and increased CSF testosterone and androstenedione, which might be generated by the truncal adipose tissue rather than the ovaries [74]. On the other hand, limited evidence suggests that men with testosterone deficiency might be more prone to developing IIH [78], leading to the inference that a certain range of testosterone concentrations, achievable by both hyperandrogenic females and hypogonadal males, favors visceral fat deposition and IIH [68].

While the role of growth hormone also warrants consideration [79], the role of estrogens in IIH remains largely unexplored, since estrogen receptors are expressed by choroid plexus cells, but data regarding estrogen concentrations in the CSF are inconsistent [52,80].

Furthermore, other pathogenic hypotheses linking obesity to IIH have been proposed. Firstly, higher concentrations of the chemokine CCL2 [81] alongside IL-2 and IL-17 [82] in the CSF compared to controls have been found in obese subjects with IIH, suggesting inflammatory mechanisms.

Secondly, the enzyme 11-beta-hydroxysteroid dehydrogenase (11β-HSD1), which is highly active in fat tissue and correlates with metabolic issues [83], is also expressed in the choroid plexus [84], where it stimulates epithelial sodium transporters, potentially increasing CSF production [83]. Therapeutic weight loss reduces 11β-HSD1 activity and, in parallel lowers ICP, paving the way for investigations regarding the efficacy of 11ß-HSD1 inhibitors in IIH [85].

Thirdly, leptin levels have been found to be higher in both the serum and CSF of individuals with IIH compared to BMI-matched controls [86,87], suggesting unimpaired leptin transport across the blood–brain barrier [87]. Moreover, given the absence of increased leptin-induced hypothalamic satiety, hypothalamic leptin resistance has been postulated [88].

Fourthly, a higher prevalence of obstructive sleep apneas (OSAs) has been reported in patients with IIH of both sexes [89,90]. Likewise, evidence from both adult [90] and pediatric [91] studies suggests that resolution of OSA can lead to improvements in papilledema, visual symptoms, as well as CSF opening pressure (OP), even independent of BMI changes. The mechanisms underlying the association between OSA and IIH could involve cerebral vasodilation and increased cerebral blood flow secondary to nocturnal hypoxia and hypercarbia and/or elevated intrathoracic pressure at the end of apnea. In keeping with this, it has been proposed that carbonic anhydrase inhibitors could also alleviate IIH by enhancing respiratory drive through metabolic acidosis [92]. Alternative mechanisms driven by OSA could include disruption of the blood–brain barrier, a hypercoagulability state, impaired venous drainage due to fat deposition both in the neck and in the chest, and glutamate-induced neuro-excitotoxicity [92]. However, recent research has questioned the pathogenic role of hypercarbia, highlighting the need to further clarify the need for routine screening for OSA as well as the role of its treatment in patients with IIH [93].

Finally, the glymphatic system (GS), which plays a key role in clearing brain waste via CSF, has been implicated in the pathophysiology of IIH [5,6]. Its dysfunction, exacerbated by obesity and metabolic syndrome, impairs the clearance of neurotoxic metabolites and promotes neuroinflammation [5,6]. Animal studies also showed that altered expression of Aquaporin 4 (AQP4), a water channel on astrocytes, reduces glymphatic flow, contributing to raised ICP and cognitive decline [5,6].

## 7. Current and Emerging Strategies for Pediatric IIH Management

Due to the lack of prospective and randomized trials in childhood and adolescence, current treatment options for IIH in this age group are borrowed from adults [2,3].

Recently, a Delphi consensus was developed in the UK to help standardize the management of pediatric IIH [94]. The main goal of treatment is to lower intracranial pressure (ICP) to relieve symptoms and to identify any underlying causes [2,3,34].

Because of its complexity, IIH requires a multidisciplinary approach. The team usually includes a neurologist, neuro-radiologist, ophthalmologist, orthoptist, nutritionist, neurosurgeon, and pediatrician. A pediatric endocrinologist should be involved if there are concerns about excessive weight or other endocrine issues that might be contributing to the condition.

The choice of treatment depends on the severity of the symptoms and the availability of specialized medical teams. The initial approach typically combines weight loss strategies with medication.

### 7.1. Non-Pharmacological Weight Loss Strategies

Weight loss is the only established disease-modifying therapy in obese subjects with IIH [2,3,72,95]. It has been reported that a weight loss of at least 6% to 10% can allow a significant reduction in the signs and symptoms of IIH [50,95,96]. It has also been reported that truncal weight loss may be specifically associated with IIH remission [68]. So far, the best weight loss strategy, as well as the amount of weight loss required, remains uncertain. Improvements in IIH symptoms were observed with a nutritionally complete low-calorie diet that induced around 15% weight loss [97]. This included a substantial drop in ICP, improvement of papilledema, and a 50% reduction in headache frequency and severity, along with less analgesic use [97]. Despite being frequently suggested, a low-salt diet has minimal supporting evidence in this patient group [95]. The Idiopathic Intracranial Hypertension Treatment Trial (IIHTT) employed a multicomponent, year-long outpatient program aimed to achieve weight loss through a combination of dietary, exercise, and behavioral strategies [98]. Patients followed a balanced (20% protein, 25–30% fat, remainder carbohydrates) low-sodium diet, and received comprehensive nutrition competency training. The exercise component comprised 30–60 min of moderate-intensity exercise or 20–60 min of vigorous-intensity exercise daily for at least five days a week. Cognitive behavioral therapy addressed goal setting, relapse prevention, and healthy lifestyle habits [98]. The group that received the dietary intervention showed a mean weight loss of just 3.5 kg (3.5%), as compared to the group receiving also acetazolamide, which showed a mean weight loss of 7.5 kg (7.8%) over 6 months [98].

Bariatric surgery leads to greater and more sustained weight loss compared to dietary regimes [99] and is being increasingly suggested as a lasting therapy to induce IIH remission in non-compliant patients [99]. In addition, recent studies with a follow-up of at least two years indicate positive effects on ICP reduction greater than that observed with conventional therapy [100,101]. The results of a recent systematic review of seventeen adult studies confirmed that bariatric surgery showed the most significant weight loss and the largest reduction in ICP in adults, in comparison to multicomponent lifestyle programs with acetazolamide or very low-energy diets. A strong correlation was found between body weight reduction and ICP reduction [102]. According to the recent clinical guidelines of the American Academy of Pediatrics, bariatric surgery can be considered in adolescents above age 13 years, who have severe obesity (BMI over 35 kg/m^2^ or 120% of the 95th percentile for age/sex) and moderate to severe comorbidities, including IIH [103]. So far, data regarding the effects of bariatric surgery in children with IIH are anecdotal. A recent case report of a 16-year-old adolescent undergoing laparoscopic sleeve gastrectomy reported reduced bilateral papilledema five months post-surgery [104]. By 18 months, he had lost 67.5% of his excess weight, and all IIH symptoms had fully resolved [104].

### 7.2. Traditional Pharmacotherapy

Because IIH is poorly understood and its cause is unknown, there exists an unmet need for effective, targeted treatments. Indeed, most currently available drugs (Table 3) simply aim to reduce ICP by decreasing CSF secretion [2,3]. Acetazolamide, a carbonic anhydrase inhibitor, is the mainstay of treatment in children [2,3]. It has shown effectiveness in relatively large pediatric studies, where clinical benefits were reported in up to 76% of the subjects treated [105]. Patients who were younger at presentation were less likely to respond [105]. Nevertheless, a 2015 Cochrane review found insufficient evidence to support its efficacy in treating the condition [106]. Notably, acetazolamide did not impact headache in the IIHTT study [98]. Similarly, while topiramate has shown a significant in vivo effect in reducing ICP [107], a recent open-label trial found that it only provided a marginal reduction in ICP, with no significant difference compared to other commonly used drugs [108].

### 7.3. Beyond Symptomatic Relief: Emerging Pathophysiology-Targeted Therapies for IIH

In addition to traditional treatments, new and promising strategies targeting obesity-related mechanisms are emerging. In particular, in recent years, research has focused on treatment strategies, which may have a pathophysiological rationale, being targeted at obesity-related neuro-metabolic disturbances (Table 3).

11β-HSD1 inhibitors lower local cortisol in the choroid plexus, which in turn reduces sodium and water transport and decreases CSF secretion [83,84,85]. A Phase II trial of the inhibitor AZD4017 showed a significant decrease in ICP without affecting systemic glucocorticoid metabolism [85]. Additionally, AZD4017 improved lipid profile, liver function, and increased lean body mass, despite not affecting BMI or fat mass [109].

GLP-1 receptor agonists (RAs) are currently approved for type two diabetes and as a body weight lowering treatment in obese patients [110,111]. GLP-1 is a gut hormone released after a meal, regulating blood glucose metabolism. It is also produced by neurons in the nucleus tractus solitarius that extend to the hypothalamus, where it promotes satiety and weight loss [112]. Finally, GLP-1 acts as a diuretic by increasing sodium and water excretion in the kidneys. Given that similar fluid transport mechanisms exist in the choroid plexus (where GLP-1 receptors are also expressed), a direct influence of GLP-1 on CSF dynamics has been hypothesized [113,114]. The GLP-1 agonist exendin-4 effectively reduced ICP in rats within 30 min [115] and with a cumulative effect over several days [116]. In keeping with this, in a study of 16 women with active IIH, subcutaneous exenatide (a synthetic form of exendin-4) significantly lowered ICP at 2.5 h, 24 h, and 12 weeks, with no serious safety concerns [117]. More recent large-scale retrospective real-world data indicate that liraglutide provides sustained risk reduction for papilledema over two years [118], whereas the addition of semaglutide to standard IIH management may reduce the risk of visual disturbances, papilledema, headache [110] and refractory disease over a 24-month period [119]. GLP-1 RAs users also seem to require less acetazolamide use and fewer shunt placements [110]. Similar positive effects have also been observed for the dual GIP/GLP-1 receptor activator tirzepatide currently approved only in adults, as recently analyzed in real-world study by Azzam and co-workers [118].

Octreotide has been hypothesized to manipulate CSF secretion due to the presence of somatostatin receptors on the choroid plexus [116,120,121]. A prospective study involving 26 patients found resolution of papilledema in 92% of cases, even though the lack of a control group limits interpretation of these results [116].

Finally, a growing body of evidence suggests that restoring glymphatic function may help counteract neuroinflammation associated with obesity and metabolic disease, as recently reviewed by Chen et al. [6]. *Alisma orientale* has been shown to improve glymphatic clearance by restoring AQP4 expression in the hippocampus, reducing neuroinflammation, and improving blood–brain barrier integrity in high-fat diet-fed mice [6]. Similarly, bafilomycin A1, a lysosomal inhibitor, prevents AQP4 degradation and partially rescues cognitive function in diabetic models [6]. MMP9 inhibitors also help maintain AQP4 polarity and perivascular integrity, which might contribute to brain fluid drainage and ICP regulation [5,6].

### 7.4. Surgical Options

Surgical options should be considered in cases with progressive vision deterioration (e.g., worsening visual acuity, visual fields, or papilledema grade) despite maximally tolerated medical management or severe and rapidly advancing vision impairment [9,11]. CSF diversion procedures, including lumboperitoneal or ventriculoperitoneal shunt, represent the most used surgical option [9,11,122]. Optic nerve sheath fenestration (ONSF) may be preferred over shunting if the primary concern is vision [9,11], or can be chosen if a shunt is not feasible or fails [9,11]. Finally, venous sinus stenting (VSS) might be considered in patients who have a significant stenosis of the dural venous sinus (most commonly in the transverse sinus) with a clear venous pressure gradient documented by manometry, or have unsuccessfully undergone, declined, or are unsuitable candidates for other surgical procedures [8].

## 8. Treatment Monitoring

There is no standardization about follow-up of IIH during childhood and adolescence [8,9,14,94].

Close follow-up is essential to detect any changes in a patient’s vision and symptoms [6,9,13]. Patients should undergo a complete eye exam at baseline, including visual fields (by using Humphrey or Goldmann method, as appropriate for age), color vision, visual acuity, OCT, and B-scan. The frequency of these visits, initially ranging from weekly to monthly for some children, will be adjusted based on the severity of papilledema and vision, eventually extending to every 1 to 3–6 months [6,123].

Depending on the objective set, lifestyle intervention programs may be of moderate-intensity, comprising one to two treatment sessions monthly, or of high-intensity, providing at least 14 sessions over 6 months [98]. Patient motivation could be periodically enhanced by informing them that even a modest (6–10%) weight loss may be enough to improve IIH symptoms. Furthermore, it is important to convey that recurrence often follows a comparable weight gain [20,98,124]. Ongoing support and counseling from a dietitian or healthcare provider can help patients adhere to healthy eating patterns and maintain lifestyle changes [98,124].

The necessity of routine blood tests for patients on acetazolamide is debated. While the Food and Drug Administration (FDA) [125,126,127] and a recent Delphi consensus for pediatric IIH [94] both recommend periodic monitoring of urea, electrolyte, and bicarbonate levels and full blood counts, with the consensus specifically advising that bicarbonate levels should be corrected if they fall to 18 mmol/L or lower [94], some studies [105,123] have found that routine testing may be unnecessary.

Particular attention should be paid at monitoring patients taking GLP-1 RAs, due to the high rate of adverse drug reactions, especially gastrointestinal [125,126]. These patients require tight and tailored monitoring, especially during the initial dose escalation phase, in order to assess response to medication, effectiveness in achieving treatment goals and make any necessary adjustments to the dosage [125,126]. If a patient does not achieve at least a 5% weight loss after 6 months of treatment on the maximum tolerated dose, discontinuation of semaglutide may be considered [126].

Finally, patients who qualify for bariatric surgery, should be looked after by specialized teams and follow a strict follow-up protocol after the operation, aimed at monitoring the efficacy and complications (e.g., symptomatic gallstone disease and small bowel obstruction) of the specific procedure [125]. Bariatric surgery may also carry the risk of micronutrient deficiencies, including iron, B vitamins, vitamin D, vitamin A, and folate, thus requiring regular supplementation [125]. In addition, adolescents may exhibit a reduction in bone mineral density [125].

## 9. The Impact of Obesity on Long-Term Outcomes of IIH

Prospective data on the natural history of IIH in children and adolescents are scanty. Current literature indicates that approximately one-third of patients may experience a relapsing disease course, highlighting the importance of long-term surveillance [40,42,128]. The rate of recurrence is estimated at approximately 10% in the first year and then rises to about 20% in the second and third years [42]. Prepubertal children seem to relapse sooner than older patients [128]. Notably, the risk for IIH recurrence is five times higher in children with overweight or obesity, compared to normal weight [129]. Moreover, an increase in body weight of 6% since initial resolution has been shown to increase the risk of recurrence in adult women [129]. Existing studies also suggest the epidemic of childhood obesity will lead to increased morbidity from IIH. Indeed, in a recent retrospective study involving 134 IIH patients with severe papilledema, the patient with worse visual outcome (defined as poor visual acuity and constricted visual field in at least one eye) had significantly higher BMI than patients with good outcome [130]. Puberty also correlates with worse visual outcomes, suggesting a potential influence of hormonal factors on disease severity [131].

The psychosocial impact of IIH is increasingly recognized as a significant component of disease burden. Individuals with IIH exhibited significantly higher levels of anxiety and depression, suggesting that factors beyond obesity contribute to the impairment of Health-Related Quality of Life (HRQoL) in this population [132]. Further on headache has emerged as a critical determinant of reduced HRQoL [133], depression and anxiety in IIH [134]. Such comorbidities, in turn, can impact negatively on weight loss, making the management of IIH more difficult [102].

Adding to these effects, a large cohort of women with IIH showed a more than twofold increased risk of cardiovascular disease (CVD) and type 2 diabetes compared to BMI-matched controls [18]. This suggests that the risk is not solely explained by obesity, but that IIH is associated with a broader metabolic dysregulation [18].

## 10. Conclusions and Future Directions

Our review extends beyond prior comprehensive works, by incorporating recent epidemiological data and emerging therapeutic strategies for IIH. This is all the more significant in the wake of the worldwide obesity epidemic and the increasing use of GLP-1 agonist medications. Crucially, this review distinctly focuses on the paediatric and adolescent population with obesity-related IIH, allowing for an in-depth interpretation of age-specific challenges and the unique neuro-endocrine and inflammatory pathways linking childhood obesity and puberty to IIH pathogenesis. Indeed, recent literature has strongly reinforced the role of central obesity and glymphatic system dysfunction as key pathophysiological mechanisms.

Notably, the association between obesity and IIH suggests that IIH may be preventable. Tracking IIH incidence trends is therefore a critical metric of the effectiveness of public health initiatives, especially those designed to combat childhood obesity. Furthermore, a tailored, multidisciplinary approach and long-term surveillance are also essential to address the disease’s relapsing nature and long-term implications, including cardiovascular and metabolic risks.

Despite weight loss being the mainstay of IIH treatment, critical gaps exist in our knowledge, especially concerning the exact amount of weight loss required or the most effective weight loss strategies. Future studies on weight management interventions must prioritize long-term data over short-term results, with a specific focus on measuring patients’ quality of life.

Traditional treatment options primarily aim to reduce ICP by decreasing CSF secretion. Nevertheless, research is now exploring novel targeted therapies, especially 11β-HSD1 inhibitors, GLP-1 RAs and bariatric surgery, which address underlying metabolic dysregulation. Given that GLP-1 RAs and bariatric surgery may be already considered in adolescents with refractory morbid obesity, further studies evaluating the efficacy of these therapeutic options in this age group are necessary. Shared monitoring protocols are also desirable.

## Figures and Tables

**Table 1:** Epidemiological studies involving children or adolescents with idiopathic intracranial hypertension.

Author, Year, [Ref.]	Geographical Area	Study Design	Reference Population/Database	Age Range (Years)	Diagnostic Criteria for IIH	N. of IIH Cases	% of Females	Incidence Per Year	% of Overweight/Obese	Incidence Per Year in Obese Patients	Other
Tibussek et al., 2013, [19]	Germany	Prospective surveillance among all 368 paediatric hospitals and departments in Germany between January and December 2008	13 million	<18	Friedman	61	57.4	0.47 per 100,000	72.1% of IIH patients were obese	NA	47.54% of the IIH cases were post-pubertal
Aylward et al., 2016, [23]	United States of America, 37 countries	Registry-based retrospective study from 2003 onwards	NA/Intracranial Hypertension Registry	<18	Modified Dandy	142 (primary cases)	72.5	NA	50.4% of primary IIH patients were obese	NA	The BMI was higher in the post-pubertal group (30.7 kg/m^2^) compared to the pre-pubertal group (21.6 kg/m^2^) in females
Matthews et al., 2017, [11]	United Kingdom and the Republic of Ireland	Prospective population -based survey among general or specialised paediatricians between August 2007 and October 2009	12.451742/British Paediatric Surveillance Unit	1–16	Friedman	185	67	0.71 (0.57–0.87) per 100,000 *1–6 years* 0.17 per 100,000 *7–11 years* 0.75 per 100,000 *12–16 years* 1.32 per 100,000	81% of IIH patients were obese	*4–6 years* 0.45 per 100,000 for boys 0.56 per 100,000 for girls *7–11 years* 1.24 per 100,000 for boys 3.44 per 100,000 for girls *12–15 years* 4.18 per 100,000 for boys 10.7 per 100,000 for girls	The relative risk s associated with obesity compared to normal weight children were 3.6 and 8.1 in 7–11-year-old boys and girls, respectively and 23.3 and 26.2 in 12- 15 year old boys and girls, respectively
Nitzan-Luques et al., 2022, [8]	Metropolitan area of Jerusalem	Retrospective observational study among all three medical centers serving one metropolitan area between 2007 and 2018	400.000	<18	Friedman	82	46.34	NA	78% of IIH patients were obese	NA	67% of the IIH cases were post-pubertal
Azzam et al., 2025, [24]	United States	Retrospective observational study based on electronic health records from participating healthcare organizations between January 1990 and December 2024	51.526 (including adults)/TriNetX platform	<19	Modified Friedman	NA	NA	*4–14 years* from 14.0 (1990–1999) to 56.0 per 100,000 (2020–2024) *15–19 years* from 24 (1990–1999) to 116.0 per 100,000 (2020–2024)	NA	NA	NA

NA = not available/not applicable; BMI = body mass index; IIH = idiopathic intracranial hypertension.

**Table 2:** Pathophysiological mechanisms linking obesity to idiopathic intracranial hypertension (see text for specific references).

Pathophysiological Category	Proposed Mechanism	Key Evidence/Clinical Implications
Body Fat Distribution and Pressure	Abdominal fat increases pressure within the abdomen and chest cavity, potentially raising central venous pressure.	Abdominal fat, but not BMI, correlates with CSF OP. Therapeutic weight loss to reduce truncal fat leads to decreased intracranial pressure (ICP).
Weight Dynamics and Growth	Excess growth hormone or sex steroids production.	Moderate (5–15%) weight gain increases IIH risk in young adults (obese and non-obese). Adolescents with IIH have higher height Z-scores.
Sex Hormones	Excess androgens enhance CSF secretion by acting through androgen receptors and AKR1C3 on the choroid plexus.	Higher prevalence of PCOS in women with IIH. PCOS women with IIH show a distinct androgen profile, suggesting androgen production from abdominal fat. Men with testosterone deficiency may have higher risk of IIH.
Inflammation and CSF Metabolism	A. Inflammation: Increased cytokines and chemokines (CCL2, IL-2, IL-17) in the CSF.	Higher concentrations of inflammatory markers found in the CSF of obese subjects with IIH.
	B. Adipose Enzymes: the 11β-HSD1 enzyme (converting inactive cortisone to active cortisol) is active in fat tissue and the choroid plexus, where it stimulates sodium transporters.	Weight loss-induced reduced 11β-HSD1 activity is paralleled by ICP reduction. 11β-HSD1 inhibitors significantly decrease ICP.
	C. Leptin: Hyperleptinemia and/or hypothalamic leptin resistance.	Higher leptin levels in serum and CSF of individuals with IIH (even compared to BMI-matched controls).
Obstructive Sleep Apnea (OSA)	Nocturnal hypoxia and hypercapnia, leading to cerebral vasodilation and increased cerebral blood flow and/or intrathoracic pressure.	Resolution of OSA can improve visual symptoms and CSF OP independent of BMI changes.
Glymphatic System (GS) dysfunction	Dysfunctional clearing of brain waste via CSF.	Animal studies show altered Aquaporin 4 reduces glymphatic flow, contributing to raised ICP.

AKR1C3 = androgen-activating enzyme aldo-keto reductase family 1 member C3; 11β-HSD1 = 11β-Hydroxysteroid Dehydrogenase Type 1; BMI = Body Mass Index; CSF = Cerebrospinal Fluid; GS = Glymphatic System; ICP = Intracranial Pressure; IIH = Idiopathic Intracranial Hypertension; NA = Not Available; OP = Opening Pressure; OSA = Obstructive Sleep Apnea; PCOS = Polycystic Ovary Syndrome.

**Table 3:** Current and emerging pharmacological options for idiopathic intracranial hypertension.

Drug	Mechanism of Action	Route of Administration	Dosages	Trial in IIH Adolescents	Adverse Drug Reactions	Notes
Acetazolamide	Reduced CSF production by inhibiting carbonic anhydrase in the choroid plexus Supposed: hyperventilation secondary to metabolic acidosis, leading to improved oxygenation and reduced carbon dioxide concentrations	oral/iv	Starting dose 15 to 25 mg/kg/day, divided into two to three doses Gradual increase up to 100 mg/kg/day (maximum 2 g/day in children and 4 g/day in adolescents and adults)	Yes	Fatigue, anorexia, nausea, diarrhea, abdominal pain, altered taste, paresthesias, and altered glucose metabolism. Rare: Aplastic anemia, hypokalemia and metabolic acidosis with low serum CO_2_.	Lack of evidence in vivo suggesting significant reduction in ICP Insufficient evidence supporting its efficacy highlighted by a Cochrane review Not effective on headache
Furosemide or amiloride	Inhibition of choroid plexus carbonic anhydrase, Additional mechanisms supposed	oral/iv	*furosemide* 1–2 mg/kg/day divided in three doses or 20–40 mg three times a day (if >40 kg) *amiloride* 0.05–0.2 mg/kg/day, once daily or in two divided doses with a maximum 10–20 mg/day	Yes	Dehydration, hypokalemia, hyponatremia, hypomagnesemia, hypocalcemia dizziness, headache, nausea, vomiting, diarrhea, abdominal cramps, muscle weakness, increased blood sugar, hyperuricemia, ototoxicity, photosensitivity, allergic reactions, hepatic dysfunction, pancreatitis.	Second line treatment when acetazolamide not tolerated (or in adjunction to acetazolamide)
Topiramate	Weak inhibition of choroid plexus carbonic anhydrase Weight loss (possibly due a combination of dysgeusia or other gastro-intestinal effects plus suppression of appetite through an increase in gamma aminobutyric acid activity) Anti-inflammatory and immunosuppressant properties	oral	*6–12 years* 15 mg once daily, with an increase to a target dose of 2 to 3 mg/kg/day in two divided doses (maximum 200 mg/day) *Adolescents* 25 mg daily, with a gradual increase by adding 25 mg each week until reaching 50 mg twice daily.	Yes	Paresthesia, fatigue, dizziness, somnolence, nausea, diarrhea, weight loss, loss of appetite, speech and concentration problems, taste alteration, depression, anxiety, ciliochoroidal effusion syndrome, metabolic acidosis, rash (including severe skin reactions like Stevens-Johnson syndrome), Accelerated metabolism of drugs metabolized by CYP3A4 (ie oral contraceptives) Teratogenic effects.	In vivo evidence suggesting reduction in ICP Considered an alternative option in children who do not tolerate acetazolamide FDA-approved for neurological disorders and also, in combination with phentermine, for obesity in patients ≥ 12 years of age. Experience limited to case reports or case series with severe visual impairment
Metilprednisolone	Reduction in cerebral edema Supposed: regulation of cerebrospinal fluid dynamics	iv	High dose (15 mg/kg)	Not	Hyperglycemia, polyuria, polydipsia, weakness or fatigue, blurry vision, infections	Given in adjunction to acetazolamide until surgical treatment
Octreotide	Inhibition of GH secretion and GH receptors blockade, which can help decrease intracranial pressure Regulation of cerebrospinal fluid dynamics (somatostatin receptors expressed in the choroid plexus)	subcutaneous/iv	5–20 mcg/kg/day	Yes (limited to a few pediatric patients)		A single case report on pediatric patient
Glucagon-like peptide 1 (GLP-1) receptor agonists (RAs) or GIP/GLP-1 receptor activator	Diuretic effects, with increased sodium and water excretion in the kidneys. Improvement in excess weight, body composition, glucose and lipid metabolism, liver transaminases and blood pressure Supposed: regulation of cerebrospinal fluid dynamics (GLP-1Rs expressed in the choroid plexus), anti-inflammatory and neuroprotective properties	subcutaneous	*Semaglutide* Starting dose 0.25 mg per week. Monthly (or slower) increase in the dose up to 2.4 mg per week *Tirzepatide* Starting dose 2.5 mg per week. Monthly (or slower) increase in the dose up to 15 mg per week	Not	Nausea, vomiting, diarrhea, and constipation. Less common: acute pancreatitis, acute gallstone disease	Semaglutide approved for severe obesity above 12 years and for type 2 diabetes mellitus above 18 years Tirzepatide approved only for adults with severe obesity or type 2 diabetes mellitus
11ß-HSD1 inhibitors	Reduced CSF secretion by lowering cortisol production in the choroid plexus	oral	400 mg twice daily for 12 weeks	Not	Tiredness; hot sweats; flu like symptoms; disrupted sleep; toothache/infection; breast pain; menstrual problems; mouth ulcers; transient nausea and headaches	Experience limited to a phase II trial in adult women

NA = not available/not applicable; CSF = cerebrospinal fluid; IV = intravenous; ICP = intracranial pressure; FDA = food and drug administration.

## Data Availability

Not applicable.

## Abbreviations

The following abbreviations are used in this manuscript:

11βHSD1	11β-Hydroxysteroid Dehydrogenase Type 1
BMI	Body Mass Index
CSF	Cerebrospinal Fluid
GIP	Glucose-Dependent Insulinotropic Polypeptide
GLP-1 RAs	Glucagon-Like Peptide-1 Receptor Agonists
GS	Glymphatic System
HRQoL	Health-Related Quality Of Life
ICP	Intracranial Pressure
IIH	Idiopathic Intracranial Hypertension
MRI	Magnetic Resonance Imaging
OCT	Ocular Coherence Tomography
ONSF	Optic Nerve Sheath Fenestration
OP	Opening Pressure
OSA	Obstructive Sleep Apnea
PCOS	Polycystic Ovary Syndrome
VSS	Venous Sinus Stenting
